# The Serine/Threonine Kinase NDR2 Regulates Integrin Signaling, Synapse Formation, and Synaptic Plasticity in the Hippocampus

**DOI:** 10.1111/jnc.70094

**Published:** 2025-05-29

**Authors:** Miguel del Ángel, Atsuhiro Tsutiya, Hussam Hayani, Deniz Madencioglu, Emre Kul, Gürsel Caliskan, Yunus Emre Demiray, Alexander Dityatev, Oliver Stork

**Affiliations:** ^1^ Department of Genetics & Molecular Neurobiology Institute of Biology, Otto‐Von‐Guericke University Magdeburg Germany; ^2^ Molecular Neuroplasticity Group German Center for Neurodegenerative Diseases Magdeburg Germany; ^3^ Center for Behavioural Brain Sciences Magdeburg Germany; ^4^ Medical Faculty Otto‐Von‐Guericke University Magdeburg Germany; ^5^ Center for Intervention and Research on Adaptive and Maladaptive Brain Circuits Underlying Mental Health (C‐I‐R‐C) Jena‐Magdeburg‐Halle Germany; ^6^ German Center for Mental Health (DZPG) Site Jena‐Magdeburg‐Halle Germany

**Keywords:** hippocampus, integrin signaling, nuclear DBF2‐related kinase 2, plasticity, spatial memory, synapses

## Abstract

Nuclear Dbf2‐related (NDR) kinases are core components of the Hippo pathway, which controls neuronal polarity and neurite growth in the central nervous system (CNS). NDR2 is the principal NDR kinase in the mouse CNS, where it has been shown to regulate integrin‐dependent dendritic branching as well as growth and plasticity in hippocampal mossy fibers. Given the well‐established involvement of integrins in plasticity, we hypothesized that NDR2 might regulate synapse formation and plasticity through integrin‐mediated mechanisms. In this study, using constitutive NDR2 null mutant mice, we demonstrate that Ndr2 deficiency leads to a reduction of T788/789 phosphorylated β1 integrin expression at synaptic sites both in the hippocampal area CA1 and in primary hippocampal neurons in vitro. This reduction is associated with decreased synaptic density in both conditions and accompanied by reduced long‐term potentiation in the synapses between Schaffer collaterals/commissural fibers and CA1 pyramidal cells, which could be restored by activation of integrins with an arginine‐glycine‐aspartate‐containing peptide, as well as with mild spatial memory deficits. Together, our results suggest that NDR2 is involved in integrin‐dependent synapse formation and plasticity in the mouse hippocampus.

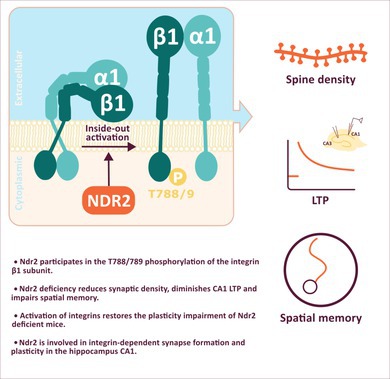

## Graphical abstract

Ndr2 participates in the T788/789 phosphorylation of the integrin β1 integrin subunit. Ndr2 deficiency reduces synaptic density, diminishes CA1 LTP, and impairs spatial memory. Activation of integrins restores the plasticity impairment of Ndr2 deficient mice. Ndr2 is involved in integrin‐dependent synapse formation and plasticity in the hippocampus CA1.

**Abbreviations**
aCSF: artificial cerebrospinal fluid
CA1: cornu ammonis 1
CA3: cornu ammonis 3
CBh: carbachol
ECM: extracellular matrix
EGFP: enhanced green fluorescent protein
EPSCs: excitatory postsynaptic currents
fEPSP: field excitatory postsynaptic potential
GRADSP: Gly‐Arg‐Ala‐Asp‐Ser‐Pro
GRGDSP: Gly‐Arg‐Gly‐Asp‐Ser‐Pro
HBSS: Hank's balanced salt solution
HFS: high‐frequency stimulation
KO: knockout
LTP: long‐term potentiation
MWM: Morris water maze
NDR: nuclear Dbf2‐related
PBS: phosphate‐buffered saline
PFA: paraformaldehyde
pβ1: phosphorylated integrin β1
ROI: region of interest
RRID: Research Resource Identifier
Stk38: serine/threonine kinase 38
Stk38l: serine/threonine kinase 38‐like
SW: sharp wave
WCM: water cross maze
WT: wild‐type

## 1 Introduction

The nuclear dbf2‐related (NDR) family of serine–threonine kinases is part of the AGC super‐family of protein kinases; they act mainly by phosphorylating downstream substrates that contain the HXRXXS/T consensus motif and core constituents of the canonical Hippo pathway (Hergovich 2016; Madencioglu 2019). The Hippo pathway has classically been implicated in various aspects of neurodevelopment, ranging from the proliferation of neural stem cells to the migration and differentiation of neuronal cells. It also plays a role in regulating synaptogenesis through actin remodeling and memory formation (Sahu and Mondal 2021; Cheng et al. 2020). In mammals, the two NDR isoforms NDR1 (*Stk38*) and NDR2 (*Stk38l*) have been implicated in neuronal development, including control of neuronal polarity and axon guidance and proliferation of neuronal progenitors (Ultanir et al. 2012; Rehberg et al. 2014; Yang et al. 2014). Previously, we showed that from the mammalian NDR kinases, NDR2 is the only one expressed in the adult mouse brain. NDR2 expression is low after birth and steadily increases during postnatal development, whereas NDR1 expression remains completely absent. Interestingly, this pattern was not observed for the rat brain, showing the importance of NDR2 for mouse neuronal biology. We showed that it controls the dendritic branching during the postnatal development of pyramidal neurons in hippocampal area CA3, growth, and plasticity of hippocampal mossy fibres (Demiray et al. 2018; Madencioglu et al. 2021). Moreover, the localization of NDR2 at synaptic sites and the induction of its gene expression during memory consolidation indicate a potential role in synaptic plasticity (Stork et al. 2004). In developing neurons, the dendritic growth effects of NDR2 are at least in part mediated by a control in the intracellular trafficking and recycling of β1 integrins (Rehberg et al. 2014; Demiray et al. 2018). Given the well‐documented relevance of integrins for synaptic plasticity and memory formation (Biose et al. 2023), it seems plausible that NDR2 may also contribute to these processes in an integrin‐dependent manner.

Integrins are heterodimeric transmembrane receptors that consist of an α and β subunits that integrate extracellular matrix (ECM) adhesion with the actin cytoskeleton (Reichardt and Tomaselli 1991) through adapter proteins such as Talin and Filamin, and intracellular signaling pathways involving various Rho GTPases, growth factor kinases, and others (Takada et al. 2007; Kiema et al. 2006). A known regulatory mechanism involves Filamin A binding to the T788/789 motif of β1 integrin, where it competes with the activators Talin and Kindlin, maintaining the integrin in an inactivated conformation. Full activation of the integrin is induced when Filamin A dissociates and Talin and Kindlin associate upon ligand binding (outside‐in signaling). This activation is further promoted by the phosphorylation of the T788/789 motif (inside‐out signaling) (Kiema et al. 2006). Filamin A is a critical factor controlling the avidity and affinity of β1 containing integrins, as it enhances their trafficking and presentation at the cell surface as well as their clustering (Truong et al. 2015). Within this signaling cascade, NDR2 appears as a key regulator by phosphorylating Filamin A at the S2152, consistent with its phosphorylation motif, thereby promoting its dissociation from the integrin tail domain (Waldt et al. 2018). β1 integrins are the predominant integrin subunit in the central nervous system. They are developmentally upegulated (Pinkstaff et al. 1999; Ikeshima‐Kataoka et al. 2022; Milner and Campbell 2002) and have been firmly implicated in neuronal development and plasticity (Wiera et al. 2022; Chan et al. 2006). Several mechanisms have been identified by which integrins may regulate synaptic biology. For instance, integrins participate in dendritic and axonal growth and regeneration (Myers et al. 2011; Fawcett 2017), spine development and reorganization by mediating interactions with the extracellular matrix and the dynamic reorganization of the intracellular cytoskeleton (Shi and Ethell 2006), and most importantly, this regulation extends to synaptic plasticity, including long‐term potentiation and various forms of memory (Chan et al. 2006, 2003).

In the current study, we investigated the potential effect of NDR2 deficiency on synaptic density in the hippocampus as well as in primary hippocampal neurons. We examined the expression of T788/789 phosphorylated β1 integrin in both conditions, as we have previously been able to demonstrate that this phosphorylation can be induced by NDR2 in developing neurons and contributes to enhanced activation of β1 integrins. Based on our observations, we further examined physiological alterations in the hippocampus, including integrin‐dependent CA3‐CA1 long‐term potentiation (LTP) and network oscillations, and probed the relevance of the observed changes in learning behavior in Ndr2 deficient mice.

## 2 Methods

### 2.1 Animal Husbandry

Stk38l^Gt(RRT116)byg^ mice (NDR2 Knockout, KO) were generated by a gene‐trap clone of the ES cell line E14TG2a, as previously described (Rehberg et al. 2014). Mutants were back‐crossed to C57Bl/6 BomTac inbred mice for more than 12 generations and were maintained as a colony with regular refreshing of Bl/6 background. Experimental mice were obtained from heterozygous breeding and genotyped at the time of weaning. Mice were maintained under an inverse 12 h light/dark cycle with a 30 min dawn phase (lights off at 7 AM). Food and water were available *ad libitum*. Mice were housed in individually ventilated cages until weaning, and then moved to standard type III cages, where they were housed in groups of 3 to 5 littermates until 3 to 4 months of age, where experimentation began. In the case of female mice used to obtain embryos for cell culture, groups of two to three females were housed together before timed breeding, where a male was introduced for 24 h.

For sample preparation, mice were exposed to isoflurane anesthesia (3% v/v). Exposure time was carefully monitored to ensure effective anesthesia while minimizing distress, and their tail flick response and paw withdrawal reflex were tested to confirm unconsciousness. Mice were scarified by cervical dislocation, and the brain or the embryos were extracted accordingly. A total of 118 animals including WT and KO mice was used for this work. Detailed information of the number of animals used for each experiment is included in each figure. Animal housing and animal experiments were performed in accordance with the European regulations for animal experiments and approved by Landesverwaltungsamt Saxony‐Anhalt (Permission Nr. 42502‐2‐1284 and ‒1712‐UniMD).

### 2.2 Study Design

For the behavioral and electrophysiology experiments, no randomization procedures were performed due to the nature of the comparisons (Ndr2 WT vs. KO). Experimenters were blinded to the genotype of the mice. No power analysis was performed, but the sample size was estimated according to similar work published by our group previously (Madencioglu 2019; Rehberg et al. 2014; Madencioglu et al. 2021; Hayani et al. 2018). All experiments were performed during the active phase of the mice, and only male mice were used for the behavioral experiments, and mice were excluded in the case that they were unable to perform tests, in the case of the reversal learning of the water cross maze (WCM) when they did not reach an accuracy of 80% during the acquisition phase or if the health of the mice deteriorated during the duration of the experiment and the distress level increased according to the guidelines for animal experimentation from the Deutsches Zentrum zum Schutz von Versuchstieren (Bf3R). Behavioral experiments were performed with independent cohorts. For the electrophysiology recordings, both male and female mice were tested in parallel in matched numbers, and data were analyzed using sex as a covariate. Irrespectively of considering sex as a covariate or not, there was no genotype‐specific difference in basal synaptic transmission and paired‐pulse facilitation, but there was a significant difference in long‐term potentiation between genotypes. Hence, the data for both sexes were pooled together and presented as such. For the in vitro quantification of spines, 2 or 3 spine segments were selected from pseudorandomized neurons located in 4 cardinal points and the center of the coverslip.

### 2.3 Home Cage Activity and Open Field Exploration

Home cage activity was monitored by infrared motion detection with an ActiMetrics detector (HCA 10‐V2.1, Coulbourn Instruments, Holliston, MA, USA) mounted over the mouse cage lid. The X, Y, and Z coordinates were tracked over 24 h under a 12 h inverted light cycle. The activity was expressed as a percentage of time spent moving over 1 h time bins. One week after monitoring the home cage activity, the mice were placed in a 50 × 50 cm square open field and left to explore for 20 min in dim white light (35 lx). 24 h later, mice were returned to the open field for another 20 min under red light (3 lx). The animals' paths were video‐tracked and the total exploration was analyzed in 5‐min bins. For the analysis of exploration patterns, the box was furthermore divided into a grid of 4 × 4 squares of 12.5 cm side each, and the time spent on the corners, the rim, or the four center squares was measured.

### 2.4 Water Cross Maze Learning

The apparatus consisted of a transparent plexiglass cross‐shaped tank. Each one of the arms had a length of 45 cm, 8 cm wide, and 30 cm‐high walls. A 10 cm tall escape platform was placed in one of the arms, and the tank was filled with water with a temperature of 20°C ± 1°C, up to 1 cm above the platform. For the training, the mice were placed in one of the arms, and access to the opposite arm of the starting position was blocked with a plexiglass wall. Four visual cues with different black and white patterns were printed on a 21 × 30 cm sheet of paper and placed outside the maze, approximately 40 cm above each of the arms for spatial orientation, correspondingly. The mice were left to swim for 30 s or until they reached the hidden platform. Each testing stage consisted of six trials with an inter‐trial interval of around 10 min every day for a period of 5 days. One week later, the location of the platform was changed to the opposite arm, and the mice underwent reversal learning following the same protocol. The latency to reach the hidden platform and the accuracy to enter the arm with the platform on the first turn were recorded and scored manually.

### 2.5 Morris Water Maze

The water maze consisted of a 150 cm diameter tank filled with water at a temperature of 20°C ± 1°C, up to 1 cm with a hidden escape platform that was placed 1 cm under the surface and 15 cm away from the edge of the tank. Visual cues with different black and white patterns were printed on 21 × 30 cm paper sheets, and then located outside the maze in the 4 cardinal points 1.6 m above the ground at an angle of approximately 45° to the centre of the tank. One day before training, the mice were habituated by placing them in the maze for five trials of 60 s in which the platform's location was visible. During training, the mice were placed inside the maze facing the border of the tank and then left to swim freely for 60 s or until they reached the hidden platform. Each testing stage consisted of five trials with an intertrial interval of around 10 min every day for 4 days in which the mouse starting location, which corresponded to each one of the cardinal points, was pseudo‐randomized. On the 5th day of training, the platform was removed for the probe trial and the mice were left to swim freely for 60 s. One week later, the platform's position was changed to the opposite quadrant, and the mice underwent reversal learning following the same protocol. The mice were recorded and the latency and time spent in the platform area during the probe trial were evaluated using the software AnyMaze.

### 2.6 Ex Vivo Electrophysiology

#### 2.6.1 Schaffer/Commissural Fibers‐CA1 Synapse Recordings

Hippocampal slices were prepared as described previously (Madencioglu et al. 2021; Hayani et al. 2018). After fast cervical dislocation, adult male mice of both genotypes (3‐ to 4‐month‐old) were decapitated and the brain was submerged into ice‐cold artificial cerebrospinal fluid (aCSF) containing (in mM): 124 NaCl, 2.5 KCl, 1.5 MgCl_2_, 1.24 NaH_2_PO_4_, 25 NaHCO_3_, 2 CaCl_2_, and 17 D‐Glucose (osmolarity of 300 ± 5 mOsm, pH 7.4). Transverse hippocampal sections with a thickness of 400 μm were prepared from dissected hippocampi using a vibrating blade microtome (Leica VT1200S, Leica Biosystems). The slices were kept in a submerged chamber in carbogen‐bubbled aCSF for at least 2 h before recording. Then, the slices were transferred into a submerged recording chamber perfused continuously with aCSF (saturated with 95% O_2_ and 5% CO_2_) at 30°C. To stimulate integrin activity, an integrin ligand containing a synthetic glycine‐arginine‐glycine‐aspartate‐serine‐proline (GRGDSP) peptide (Kramár et al. 2003) (Merck, Cat. No. SCP0157) was applied in a concentration of 0.2 mM, and a peptide containing a glycine‐arginine‐alanine‐aspartate‐serine‐proline (GRADSP) peptide (Kramár et al. 2003) (GRADSP Merck, Cat. No. SCP1056) (Kramár et al. 2003) administered in a concentration of 0.2 mM served as a control. Peptides were administered 50 min before induction of LTP, and for the whole time after induction.

The recording glass electrode filled with aCSF (2–3 MΩ) was placed in the stratum radiatum of the CA1b subregion to record field excitatory postsynaptic potentials (fEPSP) in response to Schaffer/commissural fiber stimulation. The input–output curve was obtained using 0.2 ms pulses with stimulation intensities ranging from 20 μA to 120 μA. Paired‐pulse responses were recorded using five interstimulus intervals of 10, 20, 50, 100, and 200 ms. LTP was induced using a high‐frequency stimulation (HFS) protocol containing two trains of 100 Hz (duration: 1 s, interval: 20 s) at the stimulation intensity that induced a half‐maximal fEPSP response.

#### 2.6.2 Hippocampal Network Oscillations

Horizontal brain slices (~400 μm thick) containing the hippocampal formation and entorhinal cortex were obtained as described before (Hayani et al. 2018). After fast cervical dislocation, mice were decapitated, and the brain was submerged and cut using a vibratome (Campden Instruments; Model 752) in ice‐cold aCSF containing (in mM) 129 NaCl, 21 NaHCO_3_, 3 KCl, 1.6 CaCl_2_, 1.8 MgCl_2_, 1.25 NaH_2_PO_4_, and 10 glucose (pH 7.4, ∼300 mOsm) and saturated with 95% O_2_ and 5% CO_2_. Slices were placed in an interface chamber continuously perfused with aCSF at ~32°C (flow rate: 2.0 ± 0.2 mL/min) for 1 h before commencing the recordings. For both sharp‐wave ripple and gamma oscillation recordings, glass electrodes filled with aCSF (resistance: ∼1–2 MΩ) were placed in the *stratum pyramidale* of the CA1 subregion. Analogue field potential signals were pre‐amplified and low pass filtered at 3 kHz using a custom‐made amplifier. Then, the analogue FP signal was digitized at 10 kHz using an analogue‐to‐digital converter and stored on a computer hard disc for offline analysis.

##### 2.6.2.1 Spontaneous Sharp‐Wave Ripples

Two‐min artifact‐free data were extracted as MATLAB files for further analysis (RRID:SCR_001622) as described previously (Çalışkan et al. 2022; Çalişkan et al. 2016). Data were low pass filtered at 45 Hz, and 2.5 times the standard deviation (SD) of the low pass‐filtered signal was used as the sharp wave (SW) detection threshold and minimum interval of 80 ms between two subsequent SW events. For the analysis of SW characteristics, low pass filtered data stretches of 125 ms centered to the maximum of the sharp wave event were extracted. The area under the curve (SW area) was analyzed with trapezoidal numerical integration using the points crossing the mean of the data as the start and the end of SW events. For the analysis of ripple events, the raw data were band‐pass filtered at 120–300 Hz, and a threshold of 3 times SD of the band‐pass‐filtered signal was used as the ripple detection threshold. Ripple amplitudes and frequencies were analyzed using data stretches of 25 ms centered to the maximum of the sharp wave event were extracted. The mean values of the amplitudes of the rising and falling components of a ripple were used to calculate ripple amplitude. Ripple frequencies were calculated only from subsequent ripples.

##### 2.6.2.2 Carbachol‐Induced Cholinergic Gamma Oscillations

Gamma oscillations were induced via continuous bath perfusion of Carbachol (Merck, cat. No. 212385), 5 μM at 35°C. Two‐minute data files obtained 50–70 min after Carbachol (CBh) perfusion were analyzed using custom‐made Spike2 v.8 scripts (RRID:SCR_000903). Power spectra were generated using Fast Fourier Transformation with a frequency resolution of 0.8192 Hz. Peak frequency (the frequency (Hz) at the maximum power), peak power (the power (mV^2^) at the maximum power), and integrated power (mV^2^) from 20 to 60 Hz were calculated from these power spectra. Slices with integrated powers (20–60 Hz) lower than 0.00001 mV^2^ and peak frequencies lower than 20 Hz were discarded from further analysis. Local CA1 gamma correlations were assessed with correlograms generated from corresponding low‐pass‐filtered data (< 100 Hz) using Spike2 software. The second positive peak value of autocorrelations was further used for the statistical analysis.

### 2.7 Cell Culture and Immunocytochemistry

Pregnant females were exposed to isoflurane anesthesia via passive inhalation as described before, then the tail flick response and paw withdrawal reflex were tested to confirm unconsciousness. Mice were scarified by cervical dislocation, decapitated, and the embryos were extracted. Primary neurons were obtained from the hippocampus of mice at E18.5. Around six to eight brains were obtained from each breeding event. Embryonic brains were dissected, pooled together, and neurons were disaggregated using a neural tissue dissociation kit (Miltenyi Biotec, Cat no. 130‐094‐802), according to the manufacturer's protocol. Cells were seeded in DMEM (Gibco, Cat. No. 31885049) supplemented with 10% fetal bovine serum (Sigma, cat. No 12103C), 1% Glutamax (Gibco, cat. No. 35050061), and 1% penicillin/streptavidin (Gibco, cat. No. 15070063) at a density of approximately 2.6 × 10^5^ cells per cm^2^ over cover slips coated with Poly D‐Lysine (Corning, cat. No. 734‐1102). After 24 h of incubation, media was removed and NB Media (Gibco, Cat. No. 10888022) supplemented with conditioned media (1:1), 2% B27 (Gibco, cat. No. 15717988), 0.25% Glutamax, and 1% penicillin/streptavidin was added. After 14 days in vitro, cells were fixed using 4% paraformaldehyde (PFA) in phosphate‐buffered saline (PBS), permeabilized with 0.3% Triton‐X in PBS for 10 min, and then blocked for 1 h in PBS with 5% bovine serum albumin (Sigma, cat. No. A1830) at room temperature. Cells were incubated in blocking solution with primary antibody overnight at 4°C, washed with PBS, and incubated in blocking solution with the corresponding secondary antibody. In transfection experiments, cultured hippocampal primary neurons were transfected either with a pEGFP‐C1 or a pEGFP‐C1‐Ndr2 (EGFP:Ndr2) expressing vector (Stork et al. 2004) after 10 days in vitro using lipofectamine 3000 following the instructions of the manufacturer (Invitrogen, Cat No. L3000001). Lastly, after 4 days, the cells were washed with HBSS^(−/−)^ (Gibco, cat. No. 14175095) and fixed in 4% PFA, 4% sucrose in 0.1 M PBS. Pictures were obtained using a Leica Thunder microscope (RRID:SCR_018713), and posterior image analysis was performed using Image J (RRID:SCR_003070) and Icy (RRID:SCR_010587).

### 2.8 Golgi Cox Staining

Mice were deeply anesthetized with isoflurane and subsequently decapitated to extract their brains, then a modified protocol for Golgi‐Cox staining (Glaser and Van Der Loos 1981; Mylius et al. 2013) was performed. First, the brains were immersed in Golgi‐Cox staining solution and left to incubate in the dark for 14 days at room temperature. Following impregnation, the brains underwent a series of dehydration steps using ethanol in progressively increasing concentrations (50% 3 h, 70% 10 min, 94% 24 h, 100% 48 h at 4°C) then with 100% ethanol‐diethyl ether for 4 h at room temperature, and embedded in celloidin (2% for 2 days, 4% for 3 days, and 8% for 4 days). Polymerization of the celloidin‐embedded brains was achieved through exposure to chloroform in an exicator over 1–2 days. Once polymerized, the brains were sectioned into 150 μm thick horizontal slices using a sliding microtome, with the resulting sections collected and stored in 70% ethanol. Staining of the sections involved reduction in 50% alkaline ammonia solution, enhancement with 0.5% phenylenediamine, and development in 1% DEKTOL (Kodak, cat. No. 5160270). Brain slides were fixed in 0.5% tetenal (Calbe, cat. No. 13214). Finally, the sections underwent serial dehydration with ethanol (50% 2 min, 70% 2 min, and 96% 2 min), and a solution containing 100% isopropanol, 96% ethanol (2 times 5 min), and xylol (2 times 5 min) before being mounted between coverslips using M‐GLAS (Sigma, cat. No. 10397).

For dendritic spine analysis, serial optical images of secondary dendrites from the Golgi‐stained pyramidal neurons were taken at 0.5 μm intervals in the Z‐axis (Z‐stacks) with a 100× oil objective. In the present study, we analyzed both apical and basal dendrites of pyramidal neurons in CA1 and CA3 in the hippocampus. Two to three segments approximately 50 μm length were analyzed per neuron, and four neurons were used per animal. Z‐stack images were transferred to ImageJ, and the number of dendritic spines was counted. To investigate the spine maturity state, the width and length of each spine were measured, and spines were classified into six groups, such as filopodia (length > 2 μm), long‐thin (length < 2 μm), thin (length < 1 μm), stubby (length: width ratio < 1), mushroom (width > 0.6 μm), and branched (2 or more heads) according to the method previously reported (Risher et al. 2014). Additionally, thorny excrescences were quantified in the same dendritic segments, exclusively in CA3 neurons.

### 2.9 Immunohistochemistry

Brains from 3‐month‐old mice were dissected, and the hemispheres were split using a sterile blade. The left hemisphere was immersion‐fixed in 4% PFA in PBS for 24 h at 4°C. The brains were then stored in a solution of 30% sucrose in PBS for 36 h. Coronal brain slices of 30 μm were obtained using a cryostat and stored in PBS with 2% NaN_3_. Brain slices were permeabilized with 0.3% Triton‐X in PBS for 10 min and then blocked for 1 h in PBS with 2% goat serum at room temperature. Antibodies (Table 1) were incubated in blocking solution, and pictures were obtained using a Leica Thunder microscope. For the analysis of intensity on the selected regions of interest (ROIs), we utilized the software Icy (De Chaumont et al. 2012). ROIs were generated to encompass specific areas of interest in the images, and using Icy's built‐in Intensity quantifier tool, we calculated the average intensity per square micrometer within these ROIs.

### 2.10 Western Blot

Dorsal hippocampus tissue was collected, and the samples were mechanically homogenized in Lysis buffer (1% Laurylmaltoside, 1% NP‐40, 1 mM Na3VO4, 2 mM EDTA, 50 mM Tris‐HCl pH 8.0, 150 mM NaCl, 0.5% DOC, 1 mM AEBSF, 1 μM Pepstatin A, and 1 mM NaF). Additionally, the buffer was supplemented with one tablet of Pierce protease inhibitor (Thermo, A32963). Samples were centrifuged at 13000 r.c.f. for 10 min at 4°C. The supernatant was collected and prepared for Western blot analysis by boiling it for 5 min in Sample buffer composed of (40% Glycerol, 240 mM Tris‐HCl pH 6.8, 8% SDS, 0.04% Bromphenol blue, 5% Mercaptoethanol).

Sample lysates were loaded onto an SDS polyacrylamide gel and transferred to an immobilon FL‐PVDF membrane (Millipore, IPFL00010). Membranes were blocked by incubating in Intercept blocking buffer (Licor, 927‐60001) for 1 h. Primary antibodies were prepared in Intercept blocking buffer and incubated overnight at 4°C. Protein signals were detected using either near‐infrared‐labeled secondary antibodies (NDR1) or HRP‐conjugated secondary antibodies (NDR2), according to the experiment (Table 1). Near‐infrared signals were imaged using an Odyssey scanner, while chemiluminescent detection was performed using an Odyssey FC (Licor). Signal quantification and normalization were carried out using Image Studio Lite software (LICOR). 

**TABLE 1:** Antibodies.

Antigen	Source	Company	Cat. No.	Concentration	RRID
Integrin β1 (Phospho T788/T789)	Rabbit	Abcam	Ab5189	1:400	AB_304771
Integrin β1	Hamster	BioLegend	102207	1:400	N/A
MAP2	Guinea Pig	Synaptic Systems	1888004	1:1000	AB_2661868
PSD95	Rabbit	Cell Signalling	3450	1:200	AB_2292883
Synaptophysin	Guinea Pig	Synaptic Systems	101004	1:200	AB_1210382
Rabbit IgG‐ (Alexa Fluor 488)	Goat	Abcam	Ab150077	1:1000	AB_2630356
Guinea pig IgG (Alexa Fluor 647)	Goat	Invitrogen	A21450	1:1000	AB_2535867
α‐tubulin	Mouse	Sigma Aldrich	T6199	1:10000	AB_477583
NDR1	Goat	MyBioSource	MBS626825	1:1000	N/A
NDR2	Mouse	Origene	OT14D8	1:1000	AB_2622897
Mouse IgG‐HRP conjugated	Goat	ThermoFischer	SA1‐100	1:50000	AB_325993
Mouse IgG‐IRDye 680	Donkey	LICOR	926‐68072	1:20000	N/A
Goat IgG‐IRDye 800	Donkey	LICOR	926‐32214	1:20000	N/A

### 2.11 Statistics

Statistical analysis and data visualization were performed using GraphPad Prism 9.5.1 (RRID:SCR_002798). Before statistical comparisons, the data were tested for normality using the Shapiro–Wilk test. A two‐way ANOVA with repeated measures (RM) and Fisher's LSD test were employed to assess differences between genotypes, brain areas, spine shape, WCM, OF, and Home‐cage activity behavioral experiments, unless otherwise specified. For the spine analysis, a one‐way ANOVA was used to evaluate the differences in the number of synapses in cultured neurons and a Student's *t*‐test (normal data) or Mann–Whitney *U* test (non‐normal data) was used when evaluating differences in spine density, as well as for the LTP, the hippocampal oscillations, and the behavior between the wildtype (WT) and KO mice. The alpha value was set at 0.5, and the *p* value is represented as: n/s = not significant; *p > 0.05; **p > 0.005; ***p > 0.0005; ****p > 0.0001. Outliers were tested for using a robust regression and outlier removal (ROUT) with a Q value of 0.5%.

## 3 Results

### 3.1 Ndr2 Deficiency Leads to a Reduction of Activated β1 Integrins at Synapses

**FIGURE 1:**
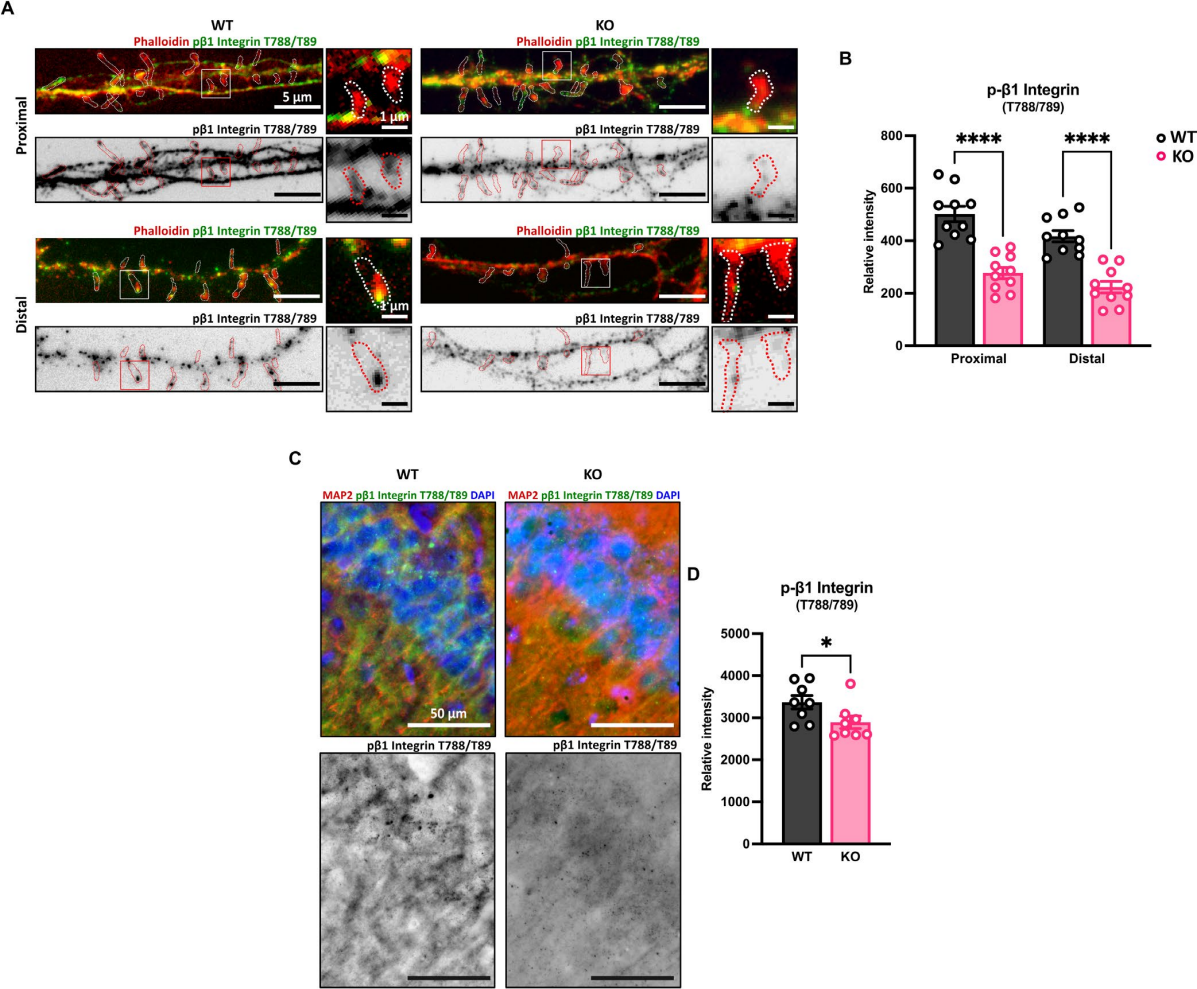
Ndr2 deficient neurons show a reduction of phosphorylated integrin‐β1 subunit intensity in spines and in the CA1 *stratum radiatum*. (A) Representative images of primary hippocampal neurons from Ndr2 wildtype (WT) and Knockout (KO) cells stained against phospho‐β1 Integrin (T788/789), green, and counterstained with Phalloidin‐Rhodamine, red. Labeled fields circumscribe individual spines selected for quantification. (B) Quantification of the phospho‐β1 Integrin signal in the spines reveals a significant reduction in Ndr2 KO mice. Each dot represents the mean intensity of the spines in three dendritic segments from independent neurons, *n* = 10 neurons each. (C) Representative image of dorsal hippocampus sections for WT and Ndr2 KO mice stained against phospho‐β1 Integrin (T788/789). (D) Quantification in the CA1 neurons confirms the reduced phospho‐β1 Integrin labeling in vivo, *n* = 8 mice each. All data are presented as mean ± SEM. Panel B: Two‐way ANOVA with Fisher's LSD test. Panel D: Student's *t*‐test. *p < 0.05; ****p < 0.0001.

We previously demonstrated that NDR2 controls integrin‐dependent neurite growth and integrin trafficking, and its expression modulates the phosphorylation levels of T788/789 on the β1 integrin subunit (Rehberg et al. 2014), a critical regulatory switch during integrin inside‐out activation (Grimm et al. 2020). To examine the role of NDR2 kinase in synapse formation, we first evaluated the levels of T788/789 phosphorylated integrin β1 (pβ1) in both cultured hippocampal neurons and adult hippocampus from Ndr2 KO mice, with a specific focus on dendritic spines (Figure 1A,C).

We analyzed the pβ1 staining intensity at spines from both proximal and distal dendritic segments (Figure 1B) and observed an overall significant decrease in pβ1 levels at the spines of KO hippocampal neurons compared to WT (*F*_1,18_ = 27.9, *p* < 0.0001), regardless of the dendritic segment location (*F*_1,18_ = 1.4, *p* = 0.247). Immunohistochemistry of the dorsal hippocampus from adult mice further confirmed the decreased pβ1 levels in Ndr2 KO mice in neurons of the CA1 area (*t* = 2.191, df = 14, *p* = 0.045). No changes were observed in total β1 staining (*t* = 0.871, df = 10, *p* = 0.404), indicating that the reduction in pβ1 staining intensity may be due to a relative decrease in phosphorylation of the β1 integrin rather than decreased protein expression (Figure S1). However, it remains possible that small changes in total β1 protein levels have remained undetected in our experiments. Further, to control for potential compensation via induction of NDR1, Western blot analysis was performed on DH samples from Ndr2 WT and KO mice (Figure S2). No NDR1 expression was detected in either genotype, showing that the absence of NDR2 does not result in protein expression of NDR1. In summary, our findings demonstrate NDR2 deficiency directly leads to reduced T_788/789_ phosphorylated Integrin β1 levels in both hippocampal dendritic spines in vitro and in the CA1 area in vivo.

### 3.2 Ndr2 Deficiency Leads to a Deficit in Synapse Number and Maturation

**FIGURE 2:**
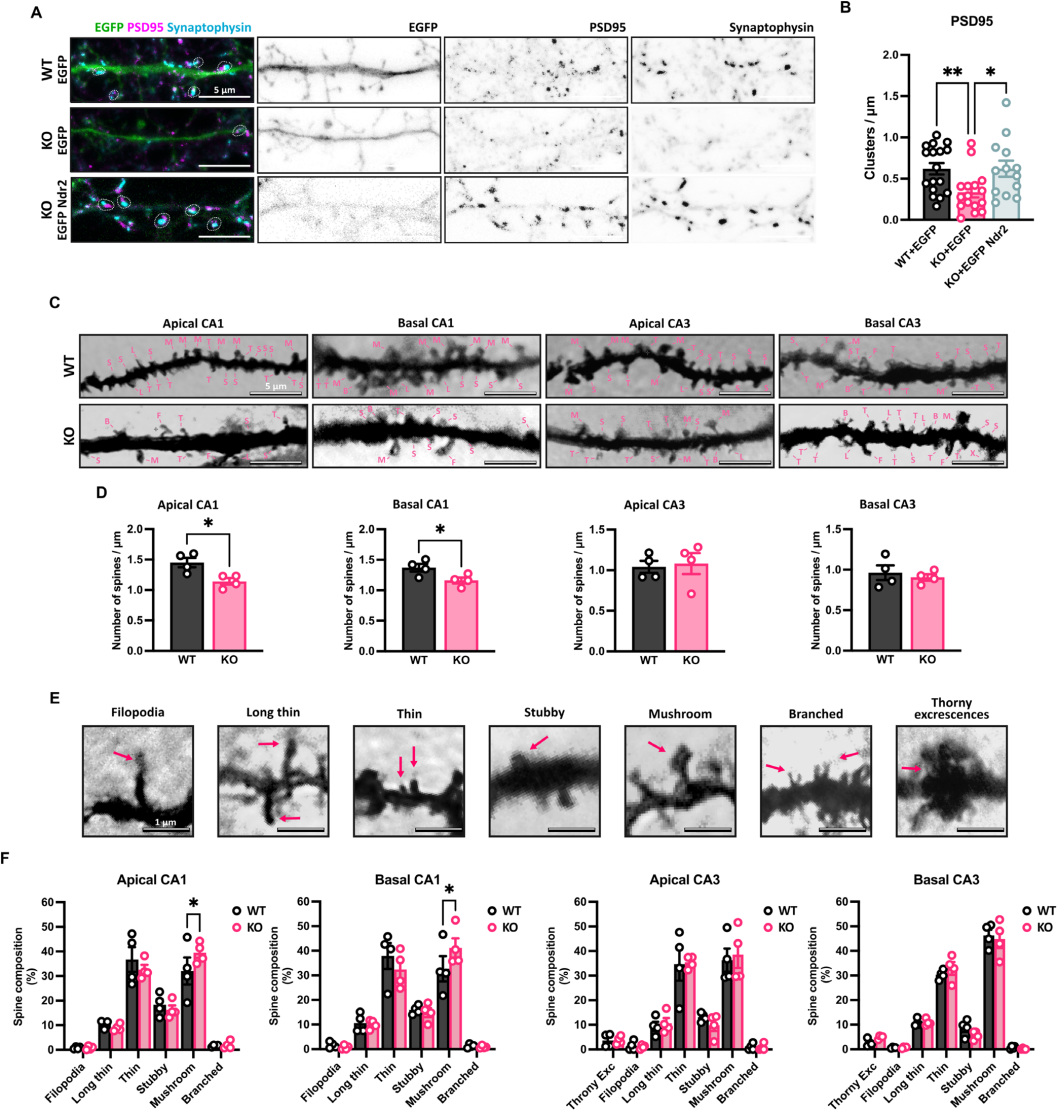
NDR2 participates in synapse formation in CA1 hippocampal neurons. (A) Primary hippocampal neurons from Ndr2 wildtype (WT) and Knockout (KO) mice transfected after 10 days in vitro with either an EGFP or EGFP:NDR2 expressing vector and stained against PSD95 and Synaptophysin after 14 days in vitro. Labeled fields circumscribe the PSD95/Synaptophysin positive puncta. (B) Quantification of PSD95/Synaptophysin positive puncta in 50 μm‐long dendritic segments reveals a significant reduction of double positive puncta in Ndr2 KO cells as compared to WT cells. KO cells are recovered to WT levels upon expression of EGFP:Ndr2. Each puncta represents the mean colocalized puncta in three spine segments from independent neurons, *n* = 17 (WT + EGFP); 16 (KO + EGFP) and 13 (KO + EGFP:Ndr2) neurons each. (C) Examples of Golgi‐Cox‐stained samples used to examine basal and apical dendrites in the CA1 and CA3 WT and Ndr2 KO mice. Arrows point out the different types of synapses quantified (F = filopodia, L = long thin, T = thin, S = stubby, M = mushroom, B = banched, X = thorny excrescences). (D) The corresponding quantification of spines in 50 μm‐long dendritic segments in these regions reveals a significant spine reduction in CA1 but not CA3; each dot represents the mean of 2 spine segments from 4 independent neurons, *n* = 4 mice. (E) Examples of spine shapes quantified: filopodia (length > 2 μm), long thin (length < 2 μm), thin (length < 1 μm), stubby (length: width ratio < 1), mushroom (width > 0.6 μm), branched (2 or more heads) and thorny excrescences. (F) A detailed analysis reveals a reduction in the proportion of mushroom spines in CA1 and an increase of thin spines in basal CA3. Each dot represents the mean of two spine segments from four independent neurons, *n* = 4 mice. All data are presented as mean ± SEM. Panel B: One‐way ANOVA with Fisher's LSD test, Panels D, E: Student's *t*‐test. *p < 0.05; **p < 0.005.

Previous research has shown that integrins play a crucial role in the formation and structural dynamics of excitatory synapses (Park and Goda 2016). Therefore, we analyzed the synaptic density of primary hippocampal neurons from WT and Ndr2 KO mice using co‐localization of Synaptophysin and PSD95 as pre‐ and post‐synaptic markers, respectively. Expression of EGFP:Ndr2 in KO neurons was performed to further control the specificity of the Ndr2 KO effects (Figure 2A). Quantification of Synaptophysin and PSD95 levels in dendritic segments (Figure 2B) revealed a significant decrease in the number of double‐positive synaptic clusters in Ndr2 KO neurons compared to the WT. Moreover, this reduction could be reversed back to control levels after NDR2 re‐expression (*F*_2,43_ = 4.927, *p* = 0.018).

Furthermore, to characterize synapses of hippocampal pyramidal cells in vivo, Golgi‐Cox staining was performed on dorsal hippocampus sections from 4‐month‐old WT and KO littermate mice, followed by analysis of spines in both apical and basal dendrites of pyramidal neurons in the CA3 and CA1 (Figure 2C). Similar to our observations in vitro, we found a reduction in spine numbers in the CA1 pyramidal neurons, both in apical (*t* = 2.605, df = 6, *p* = 0.0404) and basal (*t* = 3.299, df = 6, *p* = 0.016) dendrites. By contrast, NDR2 deficiency did not affect the spine density either in the apical (*t* = 0.276, df = 6, *p* = 0.791) or basal (*t* = 0.5882, df = 6, *p* = 0.577) CA3 neurons (Figure 2D). Additionally, spines in CA1 and CA3 were classified into six groups according to the morphological classification previously standardized by Risher et al. (2014) (Figure 2C,E); the categories included were filopodia (F), long thin (L), thin (T), stubby (S), mushroom (M), and branched (B). Additionally, thorny excrescences (X), exclusive postsynaptic elements of CA3 neurons, were also quantified. We observed an alteration in the composition of the spines. There was a significant increase in the composition of mushroom spines in both apical (*t* = 2.128, df = 36, *p* = 0.0403) and basal (*t* = 2.157, df = 36, *p* = 0.0378) dendrites in the CA1 (Fig of the KO mice (2F)). The composition of the rest of the spines, including the thorny excrescences, was not different between the WT and KO mice for any kind of spine or neuron: apical CA1 (*F*_1,6_ = 0.5226, *p* = 0.4969), basal CA1 (*F*_1,6_ = 0.3796, *p* = 0.5618), apical CA3 (*F*_1,6_ = 0.999, *p* = 0.3559), or basal CA3 (*F*_1,6_ = 0.0000, *p* = 0.9999). These results demonstrate that the deficiency in NDR2 modestly reduces spine number and alters spine maturation in the dorsal hippocampus.

### 3.3 Ndr2 Deficiency Leads to Impaired LTP in CA1 That Can Be Rescued by Integrin Stimulation

**FIGURE 3:**
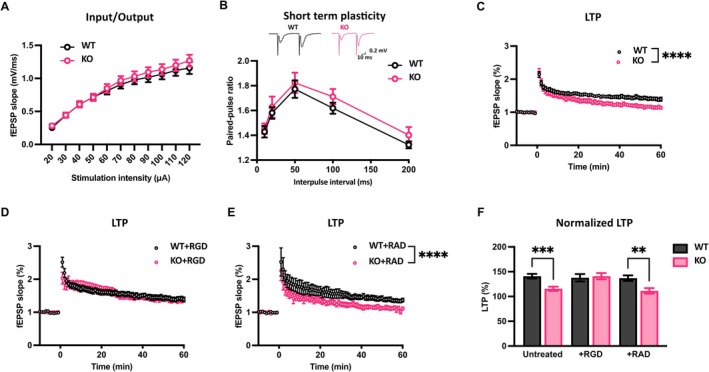
Ndr2 deficient mice display reduced synaptic plasticity in the CA3‐CA1 synapse. (A) Input–output (I‐O) curves of CA1 field excitatory postsynaptic potentials (fEPSP) slopes of Ndr2 wildtype (WT) and Knockout (KO) mice following Schaffer/commissural fiber stimulation show no differences between the WT and KO mice, *n* = 15 (WT); 12 (KO) mice. (B) Paired‐pulse ratios of fEPSP slopes in the CA3‐CA1 synapse are similar in the WT and KO mice, *n* = 13 (WT); 10 (KO) mice. (C) Normalized fEPSP slopes recorded before and after induction of long‐term potentiation (LTP) using high‐frequency stimulation (HFS) protocol in the CA3‐CA1 synapse show a profound reduction in LTP expression in hippocampal slices of KO mice, *n* = 15 (WT); 12 (KO) mice. (D) Pharmacological activation of integrins via GRGDSP peptide normalizes LTP in the KO mice, *n* = 8 mice. (E) Control GRADSP peptide does not rescue the LTP deficit in the KO mice, *n* = 8 (WT); 7 (KO) mice. (F) Summary data comparing the normalized fEPSP slopes averaged over the last 10‐min recordings of GRGDSP‐ or GRADSP‐treated hippocampal slices of WT and KO mice. All data are presented as mean ± SEM. Panels A–E: Two‐way RM ANOVA. Panel F: Two‐way ANOVA followed by Fisher's LSD. **p < 0.005; ***p < 0.0005; ****p < 0.0001.

To elucidate whether reduced phosphorylation of integrin β1 and spine density in the dorsal hippocampal CA1 is accompanied by compromised synaptic transmission and plasticity in Ndr2 KO mice, we performed ex vivo electrophysiological recordings at CA3‐CA1 synapses (Figure 3). Analysis of input–output (I‐O) curves (Figure 3A) did not reveal any difference between WT and Ndr2 KO mice in the baseline synaptic transmission (*F*_10,275_ = 0.1667, *p* = 0.9982). Similarly, we did not observe any alterations in the short‐term plasticity assessed by paired‐pulse stimulation at different stimulation intervals (Figure 3B) (*F*_4,105_ = 0.1082, *p* = 0.9794). However, we observed an impaired LTP induced by a high‐frequency stimulation (HFS) protocol in the KO mice compared to WT littermates (Figure 3C) (*F*_69,1750_ = 1.777, *p* < 0.0001). By contrast, analysis of hippocampal gamma oscillations and sharp‐wave ripples in the CA1 region revealed no differences between the naïve WT and KO mice (Figure S3). This suggests that NDR2 deficiency plays a rather selective role in modulating synaptic plasticity in this region.

Next, we elucidated whether the reduced LTP in the CA3‐CA1 synapse of KO mice may be due to the observed reduction in activated integrin levels in this region. The RGD sequence is a short amino acid motif essential for cell adhesion. It is found in various cell adhesion molecules, and its importance in recognizing and binding to integrins and the role in plasticity and LTP have been widely documented (Bahr et al. 1997; Stäubli et al. 1998; Watson et al. 2007). Treatment with the GRGDSP peptide (Figure 3D) was sufficient to restore LTP in Ndr2 KO mice (*F*_69,1190_ = 0.4652, *p* > 0.9999) while treatment with the control GRADSP peptide did not induce any rescue effect (Figure 3E). Normalization of LTP by the GRGDSP peptide was also evident when the last 10 min of LTP data were compared between different treatments, with a significant genotype × treatment interaction (*F*_2,52_ = 3.749, *p* = 0.03011) (Figure 3F). Together, these data align well with the role of integrin signaling in sustaining synaptic plasticity at the SC‐CA1 synapse (Brzdąk et al. 2023) and suggest that NDR2 is a critical component in sustaining LTP via supporting β1 integrin‐mediated signaling.

### 3.4 Ndr2 Deficiency Results in Mild Spatial Memory Deficits

**FIGURE 4:**
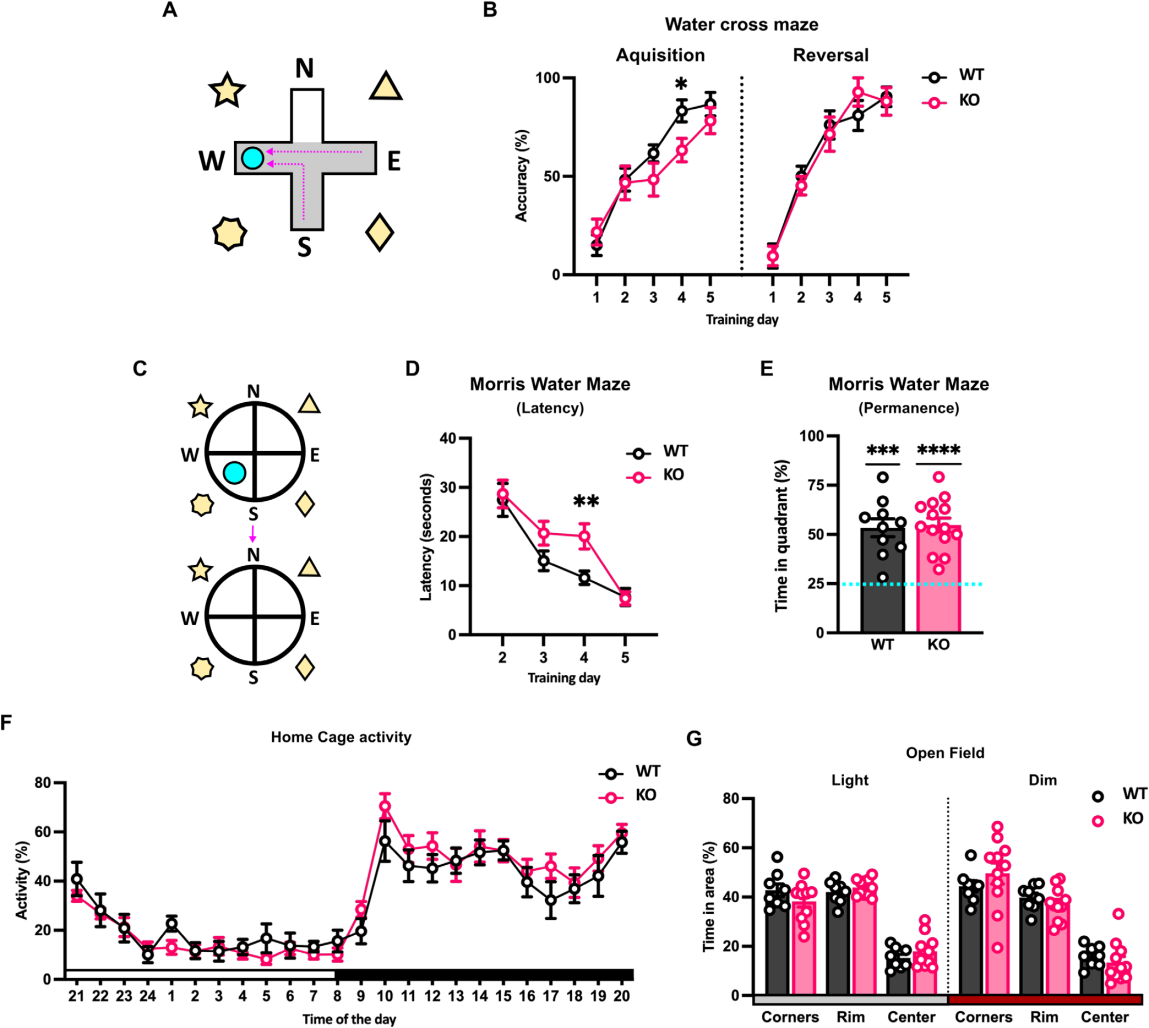
Ndr2 deficient mice display spatial memory deficits. (A) Schematics showing the Water Cross Maze (WCM) task. Adult Ndr2 KO mice and their WT littermates were trained to find the hidden platform in the WCM. One week later, the platform was changed to the opposite arm for reversal learning. (B) Ndr2 KO mice require more training to reach high accuracy levels than WT mice and showed a significant deficit on day 4. Each dot represents the average accuracy from five trials, *n* = 10 mice each. (C) Schematics showing Morris Water Maze (MWM) task. Mice were trained in the MWM for 5 days; then the platform was removed for the probe trial. (D) The latency to find the hidden platform during the training is significantly increased in Ndr2 KO mice on day 4, indicating a deficit in memory acquisition; however, WT‐like latency is achieved on the following test day during the probe trial, *n* = 11 (WT); 17 (KO) mice. (E) During the probe trial, mice of both genotypes show a significant preference for the target quadrant of the maze, *n* = 11 (WT); 17 (KO) mice. (F) No difference is evident between genotypes in baseline activity monitored in the home cage. Both WT and Ndr2 KO mice showed the expected fluctuation over light and dark periods, *n* = 8 (WT); 11 (KO) mice. (G) Exploration activity was further evaluated in an open field under two different light conditions (bright and dim), and the time spent in the corners, the rim, or the centre of the maze was quantified. No difference in exploratory activity or anxiety parameters (centre exploration) is evident between genotypes, *n* = 8 (WT); 11 (KO). All data are presented as mean ± SEM. Panels A–D, F, G: Two‐way RM ANOVA with Fisher's LSD test. Panel E: One sample *t*‐test, theoretical mean = 25%. *p < 0.05; **p < 0.005; ***p < 0.0005; ****p < 0.0001.

To address the implications of decreased connectivity and synaptic plasticity in the hippocampal CA1 area, Ndr2 WT and KO mice were tested in spatial navigation paradigms, which are known to be dependent on normal synaptic function in this region (Kleinknecht et al. 2012; Vorhees and Williams 2006). In the WCM (Figure 4A), Ndr2 KO mice displayed a decreased accuracy in entering the arm with the escape platform on the 4th day of training (*F*_12,120_ = 1.910, *p* = 0.0395) compared to the WT mice. This deficit could be overcome with another day of training in the task (Figure 4B). Moreover, when reversal training was performed 1 week later, the accuracy of finding the platform was the same between the WT and the KO mice. Similarly, in the MWM task, (Figure 4C) Ndr2 KO mice displayed a slower acquisition of the spatial location of the platform evidenced by a longer latency to reach the escape platform only on the 4th day of training (Figure 4D). Nonetheless, during the probe trial, when the platform was removed and the mice were left to swim freely for the duration of the test, no differences between the WT and KO mice were observed (*t* = 1.144, df = 26, *p* = 0.263) (Figure 4E), with both groups preferring to swim close to where the platform was located (escape quadrant). Besides, Ndr2 KO mice did not display any difference in activity, nor speed or distance traveled at any point during the day or accounting for the circadian rhythm compared to the WT mice in their home cage (Figure 4F). Moreover, we did not observe any effect on overall exploratory activity (*F*_1,17_ = 0.084, *p* = 0.111), or time spent exploring the centre area of the open field regardless of light conditions: dark (*q* = 1.140, df = 102, *p* = 9996) or light (*q* = 1.044, df = 102, *p* = 0.9998) (Figure 4G), indicating no difference in baseline activity or anxiety‐like behavior in Ndr2 KO mice.

## 4 Discussion

The Hippo signaling pathway is increasingly recognized for its importance in neuronal development and its role in several neuropsychiatric disorders such as bipolar disorder, schizophrenia (Fu et al. 2022), attention deficit hyperactivity disorder, or depression (Stepan et al. 2018). In this study, we demonstrate that one of the core kinases of the Hippo pathway, NDR2, controls the formation and maturation of hippocampal synapses as well as synaptic plasticity in the CA1 region of the hippocampus in mice. These processes are at least in part mediated by modulation of integrin functions and are associated with deficits in hippocampus‐dependent memory formation.

The NDR family comprises highly conserved serine/threonine protein kinases that play a crucial role in various cellular processes including cytokinesis, neurite outgrowth, neuroinflammation, transcription, nutrient sensing, and neuronal proteostasis (Rehberg et al. 2014; Demiray et al. 2018; Jonischkies et al. 2024). Notably, the NDR kinase homologs in *D. melanogaster* (Emoto et al. 2004) and *C. elegans* (Gallegos et al. 2004) have been shown to play critical roles in regulating dendritic tiling and branching. Furthermore, NDR1/2 has been shown to regulate dendritic growth and spine formation in rat neurons via vesicle trafficking mediated by AAK1 and Rabin8 (Ultanir et al. 2012). We have previously shown that NDR2 associates with β1 integrin‐containing early and recycling endosomes and is the dominant NDR isoform in the developing mouse brain (Rehberg et al. 2014; Stork et al. 2004). Importantly, it has been shown that impairing the endosomal integrin trafficking to the cell membrane diminishes the LTP response in the hippocampus (Babayan et al. 2012), matching the reduced hippocampal LTP in Ndr2 KO mice. Furthermore, we previously elucidated that NDR2 controls neurite growth and branching by regulating the trafficking and activity of β1 integrins in the growing neurite tips (Rehberg et al. 2014; Demiray et al. 2018). In the current study, we demonstrate the role of NDR2 in spine formation and synaptic function in the mouse hippocampus, which likely involves dysregulation of β1 integrin activity, as evidenced by reduced phosphorylated β1 integrins in NDR2‐deficient spines and the rescue of impaired hippocampal LTP through pharmacological integrin stimulation. Given that previous research has shown NDR1/2 deletions also affect interneuron maturation, further studies are necessary to determine whether NDR2 also exerts effects on the recently discovered β1 integrin‐mediated plasticity at GABAergic synapses (Wiera et al. 2022).

The integrin β1 subunit is highly expressed throughout the brain and has a widespread distribution across various neuronal populations, particularly in the dorsal hippocampus (Huang et al. 2006). Previous studies have demonstrated that β1 integrins are involved in hippocampal synaptogenesis (Warren et al. 2012; Michaluk et al. 2011) and synaptic plasticity (Shi and Ethell 2006; Bourgin et al. 2007). It is known that ECM ligands containing the RGD sequence in fibronectin or vitronectin trigger integrin outside‐in activation through binding to the cleft formed between the βpropeller and βA domains of the integrin heterodimers (Yu et al. 2014; Ruoslahti 2025). Additionally, other RGD‐derived peptides exist that are specific for different integrin subunits (Hersel et al. 2003). Peptides with a linear structure, like the GRGDSP, have higher selectivity for α5β1 integrins, in contrast to cyclical molecules that display more affinity for α5β3. Importantly, the GRGDSP peptide induces phosphorylation of the NR2A and NR2B NMDA receptors and increases NMDA receptor‐mediated excitatory postsynaptic currents (EPSCs)in an integrin‐dependent manner (Bernard‐Trifilo et al. 2005). Another publication showed that the activation of the β1 subunit using the GRGDSP is both sufficient and necessary for LTP in the CA1 (Mcgeachie et al. 2012). In summary, activation of the integrin β1 subunit using the GRGDSP seems to be critically involved in NMDA receptor‐mediated transmission and plasticity. Although we cannot rule out an involvement of other integrin subunits in the phenotype of NDR KO mice, our data thus suggest that a stimulation of β1 integrins is sufficient to rescue hippocampal plasticity in the mutants.

Besides the β1 subunit activity, α‐integrin subunits have also been heavily implicated in hippocampal plasticity. Mice deficient in both α3 and α8 integrins show deficits in LTP but not in spatial learning (Chan et al. 2010, 2007), whereas heterozygous α3, α5, and α8 deficient mice are deficient both in LTP and in hippocampal‐dependent memory, demonstrated by a decreased performance on the water maze (Chan et al. 2003). As integrin β1 can make heterodimers with a wide range of α‐integrin subunits, it appears plausible that the mild effect of NDR2 deficiency on spatial learning in our experiments may be related to a general reduction in β1 integrin activity rather than a selective deficit in a specific α/β heterodimer. It is important to note that NDR2 is involved in the trafficking of α1 subunits to growth cones and thus can affect substrate selectivity on different ECMs (Demiray et al. 2018). Even though the α1 integrin subunit has not been directly implicated in cognition or plasticity, and it is mostly studied in other organs like muscle and immune cells, it has also been shown to be expressed in the brain, particularly restricted to the pyramidal layer in the CA1 and CA3, and the granule cells in the dentate gyrus (Pinkstaff et al. 1999). Of note, α1β1 integrin heterodimer co‐localizes with the amyloid β precursor protein in axons and around astrocytes during Alzheimer's disease plaque formation (Yamazaki et al. 1997), suggesting a potential role for NDR2 in amyloidosis‐induced neuronal deficits and synaptic aging via integrin regulation. This finding aligns with the broader role of NDR kinases in aging processes, particularly in proteostasis and altered intercellular communication (Jonischkies et al. 2024).

We previously observed that NDR2 contributes to the T788/789 phosphorylation of β1 integrin cytoplasmic tail (Rehberg et al. 2014), which is known to stabilize and prevent degradation of β1 integrins (Eva et al. 2010), and as a result, increases the recruitment of β1 integrins to the cell surface, which promotes the recognition of integrin substrates such as fibronectin, ultimately enhancing neurite outgrowth (Rehberg et al. 2014). Other studies have suggested that the phosphorylation of β1 integrin at T788/789 is a critical factor for sorting in the endosomal system, as it enhances their recycling and prevents lysosomal degradation in a RAB11‐dependent manner (Powelka et al. 2004). Thus, the observed pT788/789 β1 integrin expression is likely to reflect an increased availability of activated integrins at hippocampal synapses.

While stimulation of NDR2 activity leads to T788/789 phosphorylation of β1 integrins, this phosphorylation appears to be indirect. β1 integrins do not display an NDR1/2 consensus motif, and consistently with this, experimental observations using an in vitro kinase assay targeting the cytoplasmic tail of β1 integrin as substrate have demonstrated that there is no direct phosphorylation of the β1 integrin by NDR kinases but a requirement Calcium‐calmodulin kinase II and Protein kinase C activity (Rehberg et al. 2014). Moreover, the activation mechanism of synaptic β1 integrins by NDR2 may involve the intermediate activation of other substrates. We previously demonstrated that Filamin A, which transduces mechanical signals to the cell through the actin cytoskeleton and modulation of integrin activity, is a substrate of NDR2. The mechanism involves the displacement of Filamin A from the β1 integrin cytoplasmic tail upon NDR2‐dependent phosphorylation of Filamin at Serine2152, a step crucial for the activation and stabilization of membrane integrin complexes (Waldt et al. 2018). Another possible indirect mechanism of integrin activation by NDR2 would involve the guanine exchange factor Rabin8, which increases integrin trafficking through enhanced sorting in the recycling endosomes (Eva et al. 2010) that induces neurite outgrowth (Ultanir et al. 2012; Homma and Fukuda 2016; Chiba et al. 2013). NDR2 thus may modulate integrin trafficking and activation during synaptic development and plasticity through multiple substrates.

Considering putative activation mechanisms of NDR2 at the synapse, it is evident that NDR2 kinase is very well suited to translate various development and plasticity‐related signals. MST1 and MST3 are the two most studied upstream regulators of the Hippo pathway and they can activate NDR2 kinase either through direct phosphorylation of NDR2 regulatory domains or by promoting the activity of coactivators such as MOB1 (Duhart and Raftery 2020; Stegert et al. 2005). MST1 is upregulated during spatial learning in the MWM task (Li et al. 2013) while MST3 is involved in the formation of synaptic spines and excitatory synapses (Ultanir et al. 2014). Interestingly, it has been shown that *Mst1* overexpression can induce cognitive decline and synaptic dysfunctions in an Alzheimer's mouse model (Wang et al. 2022). Another study also reported that *Mst1* overexpression disturbs hippocampal connectivity by increasing GABAergic tone and impairs spatial memory (Shang et al. 2020). Similarly, knockdown of *Mst3* reduces spine maturation in hippocampal neurons (Ultanir et al. 2014). Further upstream, another regulator of the Hippo pathway that is highly enriched in the brain is KIBRA, whose allelic variants have been associated with enhanced episodic memory performance in adults, particularly regarding the maintenance of memory during aging (Stickel et al. 2018). KIBRA can induce activation of LATS1/2 kinases and phosphorylation of the downstream effector YAP1 in the Hippo pathway (Xiao et al. 2011), but it remains to be investigated whether KIBRA can act also directly upstream of NDR2. These potential activation pathways are not unidirectional; β1 integrin signaling also feeds back to the Hippo pathway, which can negatively regulate the expression of KIBRA (Wong et al. 2016; Tokamov et al. 2021). Moreover, a recent study has demonstrated that inhibition of the Hippo pathway releases KIBRA from its association with LATS1/2, thus enhancing AMPA‐receptor‐mediated transmission (Stepan et al. 2024). Collectively, these findings highlight the critical role of finely tuned Hippo pathway regulation in cognitive function. Consequently, NDR2 kinase emerges as an intermediary player between upstream regulators such as MST kinases, MOB1, and KIBRA and downstream effectors such as integrin receptor activity that controls both synapse formation and memory processes.

Overall, we demonstrate that NDR2 kinase is involved in spine formation, synaptic function, and cognition in mice. Deficits resulting from the absence of NDR2 can be compensated for by stimulating integrin receptors or providing additional training, as observed in the MWM test. Previous studies have shown that the loss of the NDR2 homolog Trc or its regulator Fry in *Drosophila* disrupts both dendritic tiling and branching (Emoto et al. 2004) and that integrin overexpression in *Drosophila* Fry mutants rescues such tiling defects, although it fails to restore the branching phenotype (Han et al. 2012). This suggests that regulation of integrins via NDR2 plays an evolutionarily conserved role in specific aspects of neuronal morphology. The human NDR2 gene (*STK38l*) has been identified as a susceptibility gene for neurodevelopmental disorders, intellectual disability (Madencioglu et al. 2021; Firth et al. 2009), and autism spectrum disorder (Fromer et al. 2014). Additionally, *STK38l* is situated within a cryptic translocation site found in patients with Kallmann syndrome, and the ECM molecule ANOSMIN‐1, produced by the *KAL1* gene, has been demonstrated to activate β1 integrins (Choy et al. 2014; Endo et al. 2018). In conclusion, our results contribute to the growing body of evidence highlighting the significance of Hippo kinase NDR2 in integrating signals related to plasticity and cognition through the regulation of integrin signaling. The significance of NDR2 in rare genetic conditions of autism and intellectual disability, along with its potential role in modulating β1 integrins, warrants further investigation.

## Author Contributions

**Miguel del Ángel:** investigation, writing – original draft, writing – review and editing, formal analysis, visualization. **Atsuhiro Tsutiya:** investigation, formal analysis, writing – review and editing. **Hussam Hayani:** investigation, formal analysis. **Deniz Madencioglu:** investigation, formal analysis. **Emre Kul:** investigation, formal analysis. **Gürsel Caliskan:** investigation, formal analysis, writing – review and editing, methodology. **Yunus Emre Demiray:** investigation, writing – review and editing. **Alexander Dityatev:** funding acquisition, writing – review and editing, validation, supervision, resources, project administration, methodology. **Oliver Stork:** conceptualization, funding acquisition, writing – review and editing, validation, project administration, resources, supervision, methodology.

## Peer Review

The peer review history for this article is available at https://www.webofscience.com/api/gateway/wos/peer‐review/10.1111/jnc.70094.

## Supporting information


Figures S1–S3.

## Data Availability

The data that support the findings of this study are available on request from the corresponding author, O.S., A.D.
