# Supplementary material for: The Serine/Threonine Kinase NDR2 Regulates Integrin Signaling, Synapse Formation, and Synaptic Plasticity in the Hippocampus
Source: J Neurochem. 2025 May 29;169(6):e70094. doi: 10.1111/jnc.70094 (PMC12120816; doi:10.1111/jnc.70094)
Supplement: Supplementary file 1 — Figures S1–S3. [file JNC-169-e70094-s001.pdf]

The Serine/threonine kinase NDR2 regulates integrin signalling, synapse formation and synaptic plasticity in the hippocampus. Supplementary figures.

Miguel del Angel[1], Atsuhiro Tsutiya[1], Hussam Hayani[2], Deniz Madencioglu[1], Emre Kul[1], Gürsel Caliskan[1,3], Yunus Emre Demiray[1], Alexander Dityatev[2,3,4], Oliver Stork[1,3,5,6]

1- Department of Genetics & Molecular Neurobiology, Institute of Biology, Otto-Von-Guericke University, Magdeburg, Germany.

2-Molecular Neuroplasticity Group, German Center for Neurodegenerative Diseases, 39120 Magdeburg, Germany.

3-Center for Behavioural Brain Sciences, Magdeburg, Germany.

4-Medical Faculty, Otto-Von-Guericke University, Magdeburg, Germany.

5-Center for Intervention and Research on adaptive and maladaptive brain Circuits underlying mental health (C-I-R-C), Jena-Magdeburg-Halle, Germany.

6- German Center for Mental Health (DZPG), Site Jena-Magdeburg-Halle, Germany.

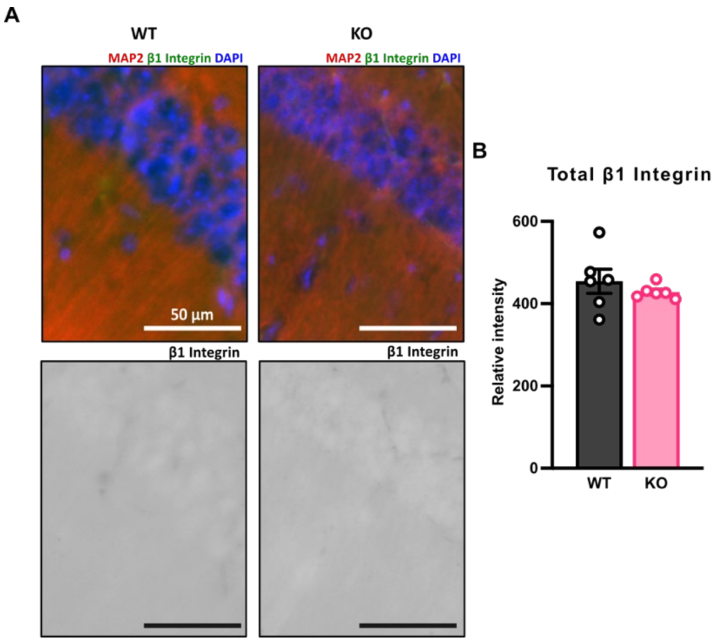

Supplementary Figure 1. Ndr2 deficient mice do not show alterations in total β1 integrin in the CA1

(**A**) Representative image of dorsal hippocampus sections for WT and Ndr2 KO mice stained against total β1 Integrin.

(**B**) Quantification in the CA1 neurons shows no changes in total β1 Integrin labelling *in vivo,* n= 6 mice each. All data are presented as mean ± SEM. Student´s t-test. *p<0.05; ****, p<0.0001.

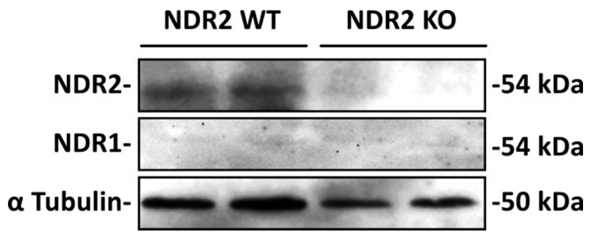

Supplementary Figure 2. Ndr2 deficient mice do not show increase in NDR1 protein expression

Representative WB of dorsal hippocampus samples of 3-month-old Ndr2 wildtype (WT) & Knockout (KO) mice against NDR1 and NDR2 n=4 (WT); 4 (KO).

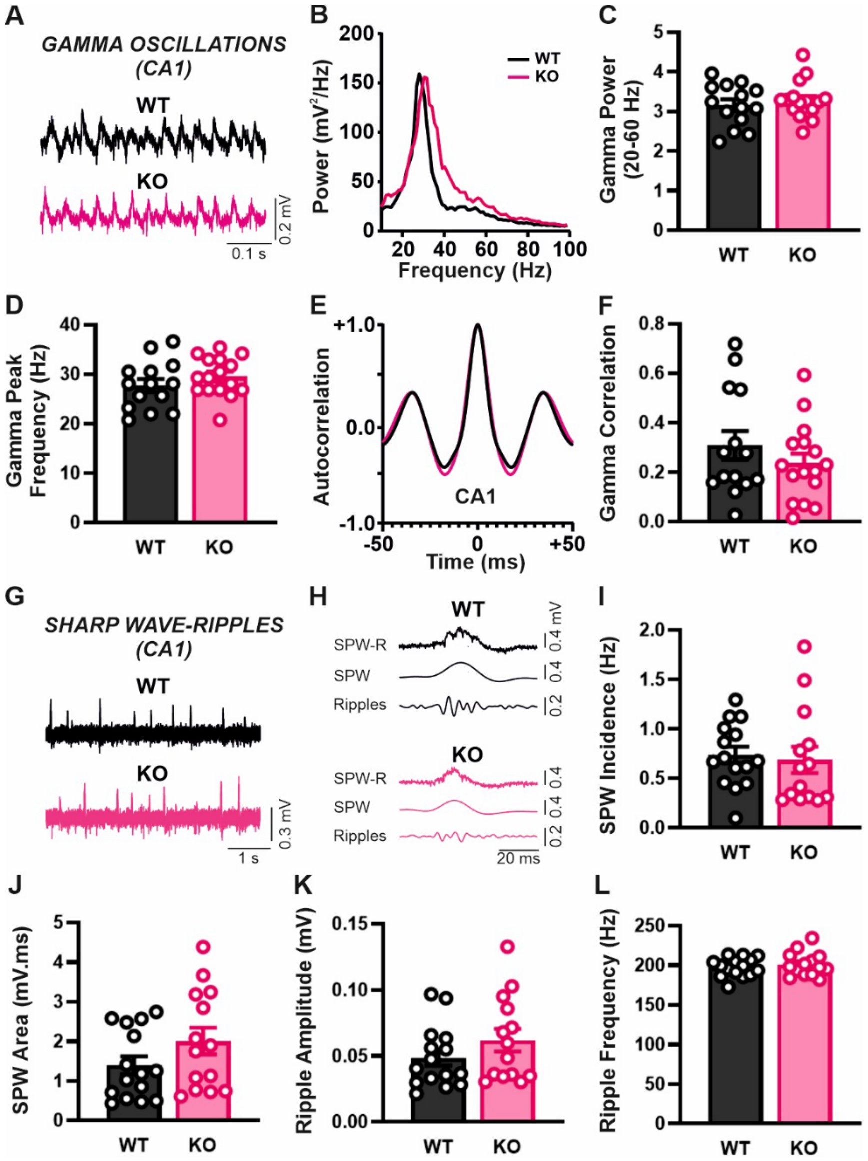

Supplementary Figure 3. Ndr2 deficient mice do not show any alterations in hippocampal network oscillations *ex vivo*.

(A) Representative carbachol-induced gamma oscillation traces from the CA1 pyramidal layer of wildtype (WT) and knockout (KO) mice, n=14 slices from 3 mice (WT), 16 slices from 4 mice (KO).
(B) Representative power spectra of CA1 gamma oscillations of WT and KO mice illustrating no changes between WT and KO mice.
(C) Summary data illustrating no changes in the integrated gamma power (20-60 Hz) (T_{28}=0.6149, p=0.5436) between WT & KO mice.
(D) Gamma peak frequency from the CA1 pyramidal neurons showing no difference between the WT and KO mice (T_{28}=1.115, p=0.2745).
(E) Representative correlograms of the gamma peak frequency.
(F) Summary data illustrating no changes in gamma correlation in the CA1 region of KO mice (T_{28}=1.073, p=0.2925).

Representative sharp wave-ripple (SPW-R) traces of WT and KO mice, n=15 slices from 4 mice (WT), 14 slices from 4 mice(KO).
(G) Low pass-filtered SPW component of WT and KO mice, n=15 slices from 4 mice (WT), 14 slices from 4 mice(KO).
(H) Band pass-filtered band-pass component of WT and KO mice, n=15 slices from 4 mice (WT), 14 slices from 4 mice(KO).
(I) Summary data of Band pass-filtered band-pass component shows no significant alterations in SPW incidence (T_{27}=0.3204, p=0.7511) n=15 slices from 4 mice (WT), 14 slices from 4 mice(KO).
(J) SPW area (T_{27}=1.537, p=0.1360)
(K) Ripple amplitude (T_{27}=1.276, p=0.2128).
(L) Ripple frequency (T_{27}=0.6739, p=0.5061).
Data are presented as mean ± SEM. Panels A, C, D, F, I, J, K and L: Student´s two-tailed T-test.
